# Loss of H3K27me3 in meningiomas: an independent marker for CNS WHO grade 2?

**DOI:** 10.1093/noajnl/vdad112

**Published:** 2023-09-02

**Authors:** Felix Behling, Peter Paßlack, Christina-Katharina Fodi, Thomas Hielscher, Jens Schittenhelm, Farshad Nassiri, Justin Z Wang, Gelareh Zadeh, Ghazaleh Tabatabai, Felix Sahm

**Affiliations:** Department of Neurosurgery, University Hospital Tübingen, Eberhard Karls University Tübingen, Tübingen, Germany; Department of Neurology and Interdisciplinary Neuro-Oncology, University Hospital Tübingen, Eberhard Karls University Tübingen, Tübingen, Germany; Hertie Institute for Clinical Brain Research, Eberhard Karls University Tübingen, Tübingen, Germany; Center for Neuro-Oncology, Comprehensive Cancer Center Tübingen-Stuttgart, University Hospital Tübingen, Eberhard Karls University Tübingen, Tübingen, Germany; Department of Neurology and Interdisciplinary Neuro-Oncology, University Hospital Tübingen, Eberhard Karls University Tübingen, Tübingen, Germany; Hertie Institute for Clinical Brain Research, Eberhard Karls University Tübingen, Tübingen, Germany; Center for Neuro-Oncology, Comprehensive Cancer Center Tübingen-Stuttgart, University Hospital Tübingen, Eberhard Karls University Tübingen, Tübingen, Germany; Department of Neurosurgery, University Hospital Tübingen, Eberhard Karls University Tübingen, Tübingen, Germany; Center for Neuro-Oncology, Comprehensive Cancer Center Tübingen-Stuttgart, University Hospital Tübingen, Eberhard Karls University Tübingen, Tübingen, Germany; Division of Biostatistics, German Cancer Research Center (DKFZ), Heidelberg, Germany; Center for Neuro-Oncology, Comprehensive Cancer Center Tübingen-Stuttgart, University Hospital Tübingen, Eberhard Karls University Tübingen, Tübingen, Germany; Department of Neuropathology, Institute of Pathology and Neuropathology, University Hospital Tübingen, Eberhard Karls University Tübingen, Tübingen, Germany; MacFeeters Hamilton Neuro-Oncology Program, Princess Margaret Cancer Centre, University Health Network and University of Toronto, Toronto, ON, Canada; Division of Neurosurgery, Department of Surgery, University of Toronto, Toronto, ON, Canada; Princess Margaret Cancer Centre, University Health Network, Toronto, ON, Canada; MacFeeters Hamilton Neuro-Oncology Program, Princess Margaret Cancer Centre, University Health Network and University of Toronto, Toronto, ON, Canada; Division of Neurosurgery, Department of Surgery, University of Toronto, Toronto, ON, Canada; Princess Margaret Cancer Centre, University Health Network, Toronto, ON, Canada; MacFeeters Hamilton Neuro-Oncology Program, Princess Margaret Cancer Centre, University Health Network and University of Toronto, Toronto, ON, Canada; Division of Neurosurgery, Department of Surgery, University of Toronto, Toronto, ON, Canada; Princess Margaret Cancer Centre, University Health Network, Toronto, ON, Canada; Department of Neurology and Interdisciplinary Neuro-Oncology, University Hospital Tübingen, Eberhard Karls University Tübingen, Tübingen, Germany; Hertie Institute for Clinical Brain Research, Eberhard Karls University Tübingen, Tübingen, Germany; Center for Neuro-Oncology, Comprehensive Cancer Center Tübingen-Stuttgart, University Hospital Tübingen, Eberhard Karls University Tübingen, Tübingen, Germany; German Cancer Consortium (DKTK), DKFZ Partner Site Tübingen, Tübingen, Germany; Cluster of excellence (EXC 2180) “Image Guided and Functionally Instructed Tumor Therapies”, Eberhard Karls University Tübingen, Tübingen, Germany; Dept. of Neuropathology, University Hospital Heidelberg, Heidelberg, Germany; CCU Neuropathology, German Consortium for Translational Cancer Research (DKTK, German Cancer Research Center (DKFZ), Heidelberg, Germany

Accurate risk stratification for meningioma patients is challenging, particularly with borderline histology between WHO grade 1 and 2. Several molecular approaches have been proposed, but cost limit their availability.

A promising marker to identify cases at higher risk of recurrence is loss of H3K27 trimethylation. However, there were insufficient data yet to incorporate it as a grading criterion into the latest WHO classification of CNS tumors 2021 (CNS5). Only 2 studies are available showing a significant association with progression-free survival, one across all WHO grades^1^ and one focusing on anaplastic cases.^2^ After the meningioma chapter for the novel WHO classification had been prepared for release, several novel studies have been published,^2–6^ supporting the prognostic impact of H3K27me3.

Yet, none of these studies provided clear data on the clinical value to distinguish “true” grade 1 from “true” grade 2 cases, that is, cases that do not fulfill the current grade 2 criteria but still tend to early recurrence.

**Figure 1.**
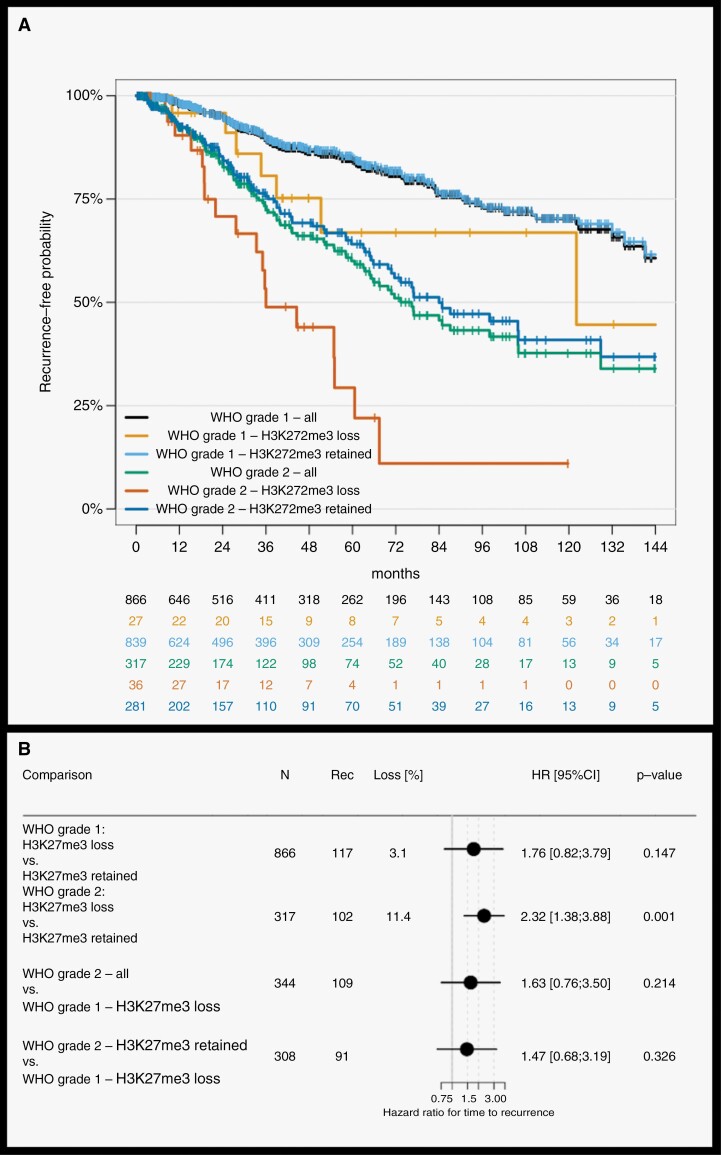
Recurrence-free survival of grade 1 and 2 meningiomas stratified for H3K27me3 status (A: Kaplan–Meier curve; B: effects in forest plot) shows a significantly higher risk of recurrence for loss within grade 2 tumors but no significant effect within grade 1 meningiomas or between grade 1 with loss and grade 2 tumors.

We therefore leveraged the 2 largest of these published studies to investigate whether H3K27 trimethylation may robustly identify morphologically grade 1 tumors with outcome identical to the average grade 2 tumors. The combined cohort comprises 866 grade 1 and 317 grade 2 primary meningiomas. Loss of H3K27me3 was observed in 3.1% (27/866 cases) and 11.4% (36/317 cases) of grade 1 and 2 tumors, respectively. A median follow-up of 39 months revealed 219 recurrences. Trimethylation loss was associated with significantly shorter recurrence-free survival (RFS) among grade 2 meningiomas, but not within grade 1 tumors. Likewise, comparing grade 1 meningiomas without trimethylation to all grade 2 tumors or grade 2 tumors with retained H3K27me3, no significant difference in clinical outcome depending on trimethylation was seen (Figure 1). Hence, even this large data set does not provide conclusive evidence for trimethylation status as grading criterion. After pooled analysis of these 2 studies that assessed trimethylation status in a binary manner, we also included data from a third cohort (Nassiri et al.). As this used a 3-tiered approach with a separate category for ambiguous cases, these ambiguous samples were excluded. Adding the remaining data to the 2 previous cohorts supported the conclusion of the previous cohorts of no clear signal for H3K27me3 status in otherwise WHO grade 1 cases (data not shown).

A limitation of all studies is enrichment for high-grade meningiomas,^1–6^ and limited follow-up for grade 1 meningiomas. Therefore, the “true” incidence of H3K27me3 loss may even be below 4.7%.^3^ Consequently, available statistical analyses are likely underpowered to show distinctions within different WHO grades: The incidence among grade 1 tumors was reported to be as low as 3.1% in 1001 tumors^3^; thus, H3K27me3 data of 1000 additional grade 1 meningiomas will likely yield 30 cases with loss. Moreover, the low rate of recurrence among grade 1 limits the statistical power for RFS. However, within grade 2 meningiomas the trimethylation loss in our data clearly shows a significantly shorter RFS. Whether such cases may even qualify for grade 3 needs to be further evaluated in comparison to a larger cohort of grade 3 tumors. While not the focus of this study, at least our data yielded no conclusive indication that grade 2 cases with H3K27me3 loss (*n* = 36) should be allotted to grade 3 (*n* = 31, HR 0.6, *P* = .1, not shown). Still, this further suggests a general biological relevance in meningioma, and that the prognostic readout may just remain statistically undetectable in grade 1 as of yet.

In conclusion, further investigation of the prognostic potential of H3K27me3 in a larger multicenter effort is necessary. Awareness of the variability of staining and standardization of assessment is warranted to derive robust conclusions from this additional data. Yet, since this immunohistochemical assay is widely established, it may be worthwhile including it in routine diagnostic workup of meningiomas and to prospectively identify and follow-up on cases with a clear H3K27me3 loss.
